# Knowledge, attitudes and practice of self-medication among university students in Portugal: A cross-sectional study

**DOI:** 10.1177/1455072520965017

**Published:** 2020-11-24

**Authors:** Regina Ferreira Alves, José Precioso, Elisardo Becoña

**Affiliations:** 155600 University of Minho, Braga, Portugal; 155600 University of Minho, Braga, Portugal; 170587 University Santiago de Compostela, Coruña, Spain

**Keywords:** knowledge, rational use of medicine, self-medication, university students

## Abstract

**Aims:**

To describe the knowledge, attitudes and practices of self-medication in college students and to analyse the predicting factors for the engagement in that behaviour.

**Design:**

This is a cross-sectional study involving students (*n* = 840) from a Portuguese university, selected through stratified and proportional sampling. Data were collected using a self-administered questionnaire containing, in addition to sociodemographic issues, a scale measuring knowledge about self-medication (α = .488), a scale measuring attitudes towards self-medication (α = .708) and questions about the patterns of self-medication practices (α = .445). Differences between outcomes and sociodemographics were analysed through independent *t*-tests and ANOVA. A generalised linear model was calculated to determine the predictive variables of self-medication.

**Results:**

Over half of the respondents ( 54.3%, *n* = 434) had used some form of self-medication during the preceding year. Students revealed poor knowledge about the referred practice, correctly answering 1.60 (*SD* = 0.936) questions in a total of 3, and favourable attitudes towards self-medication (*M* = 2.17, *SD* = 0.950, range 1–5). Attending engineering sciences (β = .718, 95% CI: 1.373–3.069, *p* < .001), being female (β = .866, 95% CI: 1.700–3.327, *p* < .001) and having negative attitudes towards self-medication (β = .367, 95% CI: 1.227–1.698, *p* < .001) predict the adoption of those practices.

**Conclusions:**

Self-medication is a common practice among university students, the level of self-medication knowledge is low and the low score of the level of attitudes revealed that students tended to have a correct positioning towards self-medication. Therefore, the recommendation to develop campaigns or educational programmes becomes obvious, in order to inform about the adverse effects of the use of non-prescribed medicine.

Self-medication is the act by which a person, on their own account or as a result of recommendation from a third party, chooses to administer medicine to themselves in order to prevent, treat or cure a condition whose identity and severity is generally unknown (WHO, 2000a). Self-medication may include using leftover drugs from treatment courses prescribed previously, or drugs obtained from relatives or friends (Ocan et al., 2015), along with “non-prescription” or “over-the-counter” medicine. In some countries, such as Portugal, non-prescription medication is not only available in pharmacies, but also in supermarkets and other outlets (Martins et al., 2016).

Self-medication is recommended by the World Health Organization (WHO) to treat self-recognised disorders or symptoms, or to treat chronic or recurring diseases or symptoms, using drugs prescribed by doctors (WHO, 2000a), making it explicitly clear that there is indeed a valid place for self-medication in developed societies. However, it simultaneously points out the need to inform the population about the appropriate use of over-the-counter medications, adopting a more educational approach to health education (WHO, 2000a). This is because the health consequences of this practice are numerous, depending on the type of medication and the varying sensitivity of each individual to them. For example, some of the repercussions for one’s health stemming from this practice include increased resistance to certain types of medication, decreased efficacy of treatments due to inappropriate use, delay of the proper diagnosis, severe medication side effects, toxicity, dangerous drug interactions, drug dependency, hypersensitivity to certain drugs, resistance withdrawal symptoms, and countless other health problems, such as drug overdose or extreme dependence (Bennadi, 2014; Hughes et al., 2001; WHO, 2000b). Nonetheless, the WHO has, however, indicated that responsible self-medication does present the advantage of preventing and treating those diseases which do not require prior medical consultation and has the potential to provide a cheaper alternative for treating common disease (WHO, 2000a).

There is an increasing tendency to use medications indiscriminately, making this practice a public health problem (Kasulkar & Gupta, 2015) in the developing countries just as much as in the already developed nations. Globally, with the exception of some North American and Northern European countries (Morgan et al., 2011), self-medication is a generalised common practice. Although the prevalence of self-medication may vary from country to country, several factors have shown a very consistent association with this practice (Carrasco-Garrido et al., 2014; Martins et al., 2002). Studies show that the main reasons for the practice of self-medication are the following: suffering from a mild illness, having previous experience in treating similar illnesses, economic conditions, unavailability of healthcare professionals and generalised excessive accessibility and availability of over-the-counter medications (Hughes et al., 2001), with young age and high educational level being frequent contributing factors for the likelihood of engaging in self-medication (Martins et al., 2002).

Several national (Mendes & Lopes, 2014; Ribeiro et al., 2010) and international (Abay & Amelo, 2010; Adhikary et al., 2014; Al Flaiti et al., 2014; Alshawi et al., 2018; Corrêa da Silva et al., 2012; Donkor et al., 2012; El-Ezz & Ez-Elarab, 2011; Galato et al., 2012; Garofalo et al., 2015; González-Castillo et al., 2019; Gyawali, 2015; Helal & Abou-Elwafa, 2017; Idris et al., 2016; Klemenc-Ketis et al., 2010; Kumar et al., 2013; López-Cabra et al., 2016; Lukovic et al., 2014; Mustafa & Rohra, 2017; Sawalha, 2008; Skliros et al., 2010; Tuyishimire et al., 2019; Xu et al., 2019) studies from various countries on self-medication practices have focused on college students, identifying an increase and a high prevalence in this particular group (Al Flaiti et al., 2014). This may be related to many factors such as sociodemographic settings, lifestyle, accessibility and availability of medication, increased knowledge, advertisement and high level of education (Martins et al., 2002).

In this study, our main aim was to describe the knowledge, attitudes and practices of self-medication among college students and to analyse the predictive factors for the engagement in that behaviour.

## Materials and methods

### Population and sample

For the 2018/2019 academic year, 5447 students were registered in the first and third years of integrated bachelor’s and master’s degrees. Excluded from the sample were courses related to health sciences, undergraduate or postgraduate masters and those that did not have classes in the first or third year. Students belonging to the area of health sciences have been excluded from this research because we felt that their knowledge about health in general had the potential to generate some degree of bias in the results of the scales used in the present study, since scientific studies indicate that students in the health area display a higher level of knowledge when compared to those belonging to other areas of study (Xu et al., 2019).

In order to carry out this study, the absolute minimum number of students we required was 592 (margin of error = 5 %, confidence level = 99 %, and response distribution = 50%). With the aim of achieving our objective, we carried out a stratified probabilistic sampling of students in the university setting based on the specific academic year they were attending and the specific scientific area of study as well. We chose to divide the several undergraduate and master’s degrees in accordance with a criterion of scientific areas attended (complying with the definition provided and issued by the Foundation for Science and Technology): social and human sciences, judicial and economic sciences, exact and nature sciences and engineering sciences.

This cross-sectional research consisting of results obtained from questioning students in the college context (*n* = 840) attending one university in Portugal has provided us with information gathered by resorting to a validated self-reported questionnaire without biochemical confirmation. The subjects who were part of the study were made up of 464 incoming students (55.2%) and 376 finalist students (44.8%). Regarding the scientific category of studies, 302 students (36.0%) belonged to the area of engineering sciences, 270 (32.1%) to the social and human sciences, 136 (16.2%) to the exact and nature sciences and 132 (15.7%) to the area of judicial and economic sciences. The majority of subjects was female (55.4%, *n* = 465), at that point not involved in a loving relationship (58.3%, *n* = 486), displaced from their usual residence (64.9%, *n* = 537), full-time students ( 88.8%, *n* = 739) and had a body mass index (BMI) corresponding to what is unanimously scientifically considered to be normal weight ( 73.1%, *n* = 599). The average age of the students who took part in this study was 20.78 years (*SD* = 4.221), with a minimum of 18 years old and a maximum of 54 years.

### Instrument

Currently, several scientific instruments exist to analyse the prevalence of self-medicating practices among young adults, such as the Youth Risk Behavior Surveillance System (YRBSS) (CDC, 2017b), the National Survey on the Use of Psychoactive Substances in the General Population (Balsa et al., 2017), the European School Survey Project on Alcohol and Other Drugs (ESPAD) (Hibell et al., 2012), the Behavioral Risk Factor Surveillance System (BRFSS) (CDC, 2017a) and the National Survey on Drug Use and Health (SAMHSA, 2017). However, none of the information gathered using the approach taken by any of these surveys entirely met the expectations and intentions behind the decision to carry out the present research. As a result of that, we developed the instruments used and referred to in this research in three different, separate stages – scale construction (first stage); content validity (second stage); and psychometric validity (third stage) – in accordance and compliance with the undertakings defined by Oppenheim (1992) and Bowling (1998).

In order to build the scale (first stage) we undertook a review of the existing literature on this topic, so that we understood which specific questions and items were commonly used in order to evaluate the level of knowledge, attitudes and practices of self-medication among students in the university academic context. We have generated an analytical matrix based on the review mentioned directed towards the different and varying dimensions we meant to analyse, and also towards identifying those with the same semantic similarities, which were excluded from the study.

For the purpose of completing content validity (second stage), 10 PhD researchers from several Portuguese universities known for the quality of their previous existing work in the area of health education and higher education have been invited to take part in a specific portion of this study. We have considered and went as far as including in the analysis data we gathered from five of the mentioned invited researchers in our final report, while all suggested semantic adaptations were also taken into account. In a similar fashion, the instrument was introduced to 12 university students, using the “thinking aloud” method (Bowling, 1998; Keszei et al., 2010) in order to identify items that might have been confusing, exclude less relevant or redundant ones, and finally to verify that pre-coded response options were sufficient. With the aim of attaining a broader and deeper level of objectivity, we have settled on the following scale as the measure of clarity for each item: 1 = confused, 2 = unclear, 3 = clear. After we received recommendations that pointed out the need to redraft, the preliminary version of the questionnaire survey was presented to a sample of 32 students who were subsequently excluded from the final sample.

The questionnaire included sociodemographic variables (sex, age, scientific area of study, academic year, weight and height (to calculate BMI), loving relationship, professional situation and current residence) and specific questions to measure the following variables:

- Prevalence of self-medication – “In the last 12 months, how many times have you consumed any of the following psychoactive substances as listed (without prescription): antidepressants/sedatives/soothing/tranquilisers; analgesics/anti-inflammatory medication; vitamins/food supplements”. Possible answers: never; 1–2 times; 3–5 times; 6–9 times; 10 or more times, with 1 point being assigned to each behaviour that was practiced at least once. Self-medication patterns were analysed according to the classification: Yes (having self-medicated at least once in the last 12 months) and No (having not self-medicated in the last 12 months). The scale’s confidence index (Cronbach’s alpha) was .445.
- Self-medication knowledge – three-item scale (“Excessive use of paracetamol causes liver toxicity”; “Changing the schedules for taking medication does not pose any danger”; “Food supplements can be taken without medical prescription because they do not cause any negative effects on the organism”). Answer options: true, false, don’t know. One point was assigned to each correct answer, while providing an incorrect answer or responding “I don’t know” resulted in 0 points. The sum of all items was calculated, hence higher scores correspond to a higher level of knowledge. Cronbach’s alpha in the sample was .488.
- Attitudes towards self-medication – two items on a five-point Likert scale (1 = strongly disagree, 5 = fully agree) (“It is acceptable to use non-prescription drugs for a short time”, and “It is acceptable to use previously prescribed medications to treat the same symptoms”). The results obtained in this scale show the following dynamics: the higher the average of the scale, the more negative the attitudes of university students toward self-medication were, ranging from 1 to 5. Cronbach’s alpha for the scale calculated with the sample of this study was acceptable (α = .708).

### Procedure and statistical analysis

The application of the instrument was carried out in the classroom context and in paper-and-pencil format for all students in the sample, after acquiring informed consent. A total number of 873 questionnaires was administered to subjects, and that number reflects the total number of students who were simultaneously present in the classroom and had accepted to take part in the study. We excluded 33 questionnaires from our final examination and results, given the fact that they were poorly filled in or given back unanswered (or answered in incomplete terms). The rate of response was 96.2% (95% CI 94.8–97.6). All ethical research procedures with humans referred to by Christensen et al. (2015) were fulfilled and the study was approved by the university ethics committee.

Data were analysed using IBM Statistical Package for the Social Sciences (SPSS), version 26.0 (Armonk, NY, USA). Descriptive analyses were performed for the demographic variables and practice questions. In order to analyse the psychometric characteristics of the scales, we have studied their reliability resorting to Cronbach’s alpha calculation (α). Regarding the topic of knowledge about self-medication and attitudes towards self-medication, the mean scores were calculated and then compared among different subgroups of respondents, using appropriate statistical tests. An independent samples *t*-test was used for dichotomous variables and analysis of variance (ANOVA) for others, with Bonferroni used for multiple comparison procedures. We calculated the connections and interactions between the results of the study resorting to Pearson’s correlation. Finally, we calculated a generalised linear model with the purpose of defining and identifying the predictive variables and factors involved in the likelihood of engaging in self-medication practices. In order to calculate this this model, we have used the sociodemographic variables which presented and showed statistically significant differences when it came to the specific matter of self-medicating practices. Betas (β) and the respective 95% confidence intervals (95% CI) are presented. A *p*-value of .05 or less was considered statistically significant, with the exception of multiple comparisons between groups, for which the Bonferroni correction was applied.

## Results

The findings showed that self-medication was a common practice among university students, since over half of the respondents ( 54.3%, *n* = 434) had used some form of self-medication during the preceding year. Analgesics/anti-inflammatories ( 40.8%, *n* = 326) were commonly used for self-medication, followed by vitamins/food supplements ( 26.4%, *n* = 211) and antidepressants/sedatives/soothing/tranquilisers ( 13.9%, *n* = 111) (Table 1).

**Table 1.** Self-medication-related characteristics.

			*f*	%
Self-medication		No	365	45.7
		Yes	434	54.3
Last 12 months	Antidepressants/sedatives/soothing/tranquilisers (without prescription)	Never	690	86.1
		1–2 times	62	7.7
		3–5 times	19	2.4
		6–9 times	8	1.0
		10 or more times	22	2.7
	Analgesics/anti-inflammatory medication (without prescription)	Never	474	59.3
		1–2 times	139	17.4
		3–5 times	94	11.8
		6–9 times	47	5.9
		10 or more times	46	5.8
	Vitamins/food supplements (without prescription)	Never	588	73.6
		1–2 times	95	11.9
		3–5 times	41	5.1
		6–9 times	19	2.4
		10 or more times	56	7.0

The results indicate that self-medication was more common among female students (62.0%, *n* = 269, χ^2^ (1) = 18.348, *p* = .000) compared to male students (38.0%, *n* = 131) (Table 2). We found that girls use more analgesics/anti-inflammatory medication (47.7%, *n* = 210) and antidepressants/sedatives/soothing/tranquilisers (19.0%, *n* = 84) than boys (32.2%, *n* = 116 (χ^2^ (1) = 19.715, *p* = .000) and 7.5%, *n* = 84 (χ^2^ (1) = 22.140, *p* = .000), respectively). Vitamins/food supplement consumption was higher in engineering science students compared to the other science areas (χ^2^ (3) = 13.652, *p* = .003).

**Table 2.** Frequencies and chi-square test for sociodemographic variables and self-medication and type of self-medication practices.

		General self-medication (Yes)		Self-medication of analgesics/anti-inflammatories (Yes)		Self-medication of antidepressants/ sedatives/ soothing/ tranquilisers (Yes)		Self-medication of vitamins/food supplements	
		*n* (%)	χ^2^	*n* (%)	χ^2^	*n* (%)	χ^2^	*n* (%)	χ^2^
Year of Frequency	First year	243 (55.4)	0.421	185 (42.1)	0.780	68 (15.5)	0.915	110 (25.1)	2.085
	Third year	191 (53.1)		141 (39.1)		43 (11.9)		101 (28.1)	
Scientific area	Engineering sciences	163 (56.6)	2.725	116 (40.3)	1.301	29 (10.0)	9.353^*^	98 (34.0)	13.652^**^
	Exact and natural sciences	77 (56.6)		58 (42.6)		27 (19.9)		29 (21.3)	
	Judicial and economic sciences	63 (55.3)		41 (44.3)		13 (11.3)		27 (23.7)	
	Social and human sciences	131 (50.2)		101 (38.7)		42 (16.1)		57 (21.8)	
Sex	Male	165 (46.0)	18.348^***^	116 (32.2)	19.715^***^	27 (7.5)	22.140^***^	92 (25.6)	0.205
	Female	269 (61.1)		210 (47.7)		84 (19.0)		119 (27.0)	
Age	< 20 years	181 (53.9)	0.047	139 (41.4)	0.092	46 (13.6)	0.021	76 (22.6)	4.283^*^
	≥ 20 years	253 (54.6)		187 (40.3)		65 (14.0)		135 (29.2)	
Loving relationship	Yes	186 (53.6)	0.190	142 (41.2)	0.020	40 (11.5)	2.795	95 (27.4)	0.254
	No	246 (55.2)		182 (40.7)		70 (15.7)		115 (25.8)	
Current residence	Displaced	157 (56.5)	0.758	112 (40.3)	0.059	46 (16.5)	2.969	71 (25.5)	0.129
	Not displaced	271 (53.2)		210 (41.2)		62 (12.1)		136 (26.7)	
Professional situation	Full-time student	381 (54.2)	0.024	286 (40.6)	0.140	96 (13.6)	0.296	179 (25.5)	3.559
	Worker / student	49 (55.1)		38 (42.7)		14 (15.7)		31 (34.8)	
BMI	Low weight	34 (60.7)	1.055	24 (42.9)	3.748	15 (26.8)	8.750^*^	15 (26.8)	0.093
	Normal weight	306 (53.7)		222 (38.9)		78 (13.6)		151 (26.5)	
	Overweight	85 (55.2)		73 (40.8)		17 (11.0)		39 (26.3)	

*Note*. Bold indicates statistically significant difference after applying the Bonferroni correction. BMI = body mass index.

*p < .05. ** *p* < .01. *** *p* < .001.

The level of knowledge about self-medication was 1.60 ± 0.936 (out of 3). In Table 3, it is observable that students in the natural and exact sciences and in the judicial and economic sciences have a higher level of knowledge about self-medication compared to students in the engineering sciences (*F*(3, 833) = 5.812, *p* = .001), and also that females had a higher level of knowledge than male students (*t*(835) = –3.699, *p* = .000).

**Table 3.** Mean, one-way ANOVA and *t*-test for sociodemographic variables and knowledge about self-medication.

		Knowledge about self-medication		
			ANOVA	
		Mean (*SD*)	*Z*	*p*
Scientific area	Engineering sciences	1.43 (0.940)	5.812	**.001**
	Exact and natural sciences	1.72 (0.849)		
	Judicial and economic sciences	1.77 (0.970)		
	Social and human sciences	1.64 (0.933)		
BMI	Low weight	1.74 (0.849)	1.873	.154
	Normal weight	1.63 (0.930)		
	Overweight	1.50 (0.970)		
			*t*-student	
			*t*	*p*
Self-medication	Yes	1.66 (0.896)	–2.279	.023
	No	1.51 (0.966)		
Self-medication of analgesics/anti-inflammatory medication	Yes	1.70 (0.901)	–2.589	.010
	No	1.52 (0.946)		
Self-medication of antidepressants/sedatives/soothing/tranquilisers	Yes	1.77 (0.839)	–2.223	.027
	No	1.56 (0.944)		
Self-medication of vitamins/food supplements	Yes	1.61 (0.935)	0.868	.386
	No	1.55 (0.923)		
Year of frequency	First year	1.66 (0.922)	2.117	.035
	Third year	1.52 (0.950)		
Sex	Male	1.47 (0.955)	–3.699	**.000**
	Female	1.70 (0.908)		
Age	< 20 years	1.63 (0.920)	0.875	.382
	≥ 20 years	1.57 (0.948)		
Loving relationship	Yes	1.59 (0.954)	–0.367	.714
	No	1.61 (0.915)		
Current residence	Displaced	1.66 (0.888)	1.371	.171
	Not displaced	1.57 (0.956)		
Professional situation	Full-time student	1.59 (0.927)	–1.279	.201
	Worker/student	1.72 (0.993)		
Total		1.60 (0.936)		

*Note.* Bold indicates statistically significant difference after applying the Bonferroni correction. BMI = body mass index.

The level or type of attitudes towards self-medication is 2.17 ± 0.950, a minimum of 1 and a maximum of 5, with the highest value corresponding to more negative attitudes. It means that students showed favourable attitudes towards self-medication, because the majority of respondents disagreed or strongly disagreed with the items (“It is acceptable to use non-prescription drugs for a short time” – 65.1% and “It is acceptable to use previously prescribed medications to treat the same symptoms” – 60.0%).

As shown in Table 4, there were significant differences based on practices of self-medication. Students who had self-medicated in the past year are those who exhibit more favourable attitudes toward self-medication practices (*t*(788) = –4.739, *p* = .000). This was also due to the type of medication used without supervision, i.e., regardless of the type of medication taken in the last 12 months, attitudes are always more favourable to analgesics/anti-inflammatory medication (*t*(789) = –3.830, *p* = .000), antidepressants/sedatives/soothing/tranquilisers (*t*(790) = –3.175, *p* = .002) and vitamins/food supplements (*t*(788) = –3.469, *p* = .001).

**Table 4.** Mean, one-way ANOVA and *t*-test for sociodemographic variables and attitudes towards self-medication.

		Attitudes towards self-medication	ANOVA	
		Mean (*SD*)	*Z*	*p*
Scientific area	Engineering sciences	2.25 (0.923)	1.378	.248
	Exact and natural sciences	2.10 (0.919)		
	Judicial and economic sciences	2.16 (1.048)		
	Social and human sciences	2.11 (0.945)		
BMI	Low weight	2.23 (0.834)	0.363	.696
	Normal weight	2.16 (0.925)		
	Overweight	2.22 (1.046)		
			*t*-student	
			*t*	*p*
Self-medication	Yes	2.31 (0.919)	–4.739	**.000**
	No	1.99 (0.952)		
Self-medication of analgesics/anti-inflammatory medication	Yes	2.32 (0.913)	–3.830	**.000**
	No	2.06 (0.956)		
Self-medication of antidepressants/sedatives/soothing/tranquilisers	Yes	2.44 (0.887)	–3.175	**.002**
	No	2.13 (0.950)		
Self-medication of vitamins/food supplements	Yes	2.36 (0.937)	–3.469	.**001**
	No	2.10 (0.942)		
Year of frequency	First year	2.11 (0.929)	–1.785	.075
	Third year	2.23 (0.973)		
Sex	Male	2.18 (0.967)	0.424	.672
	Female	2.15 (0.937)		
Age	< 20 years	2.11 (0.910)	–1.433	.152
	≥ 20 years	2.21 (0.977)		
Loving relationship	Yes	2.18 (0.974)	0.426	.671
	No	2.15 (0.933)		
Current residence	Displaced	2.17 (0.983)	0.252	.801
	Not displaced	2.15 (0.920)		
Professional situation	Full-time student	2.18 (0.947)	1.101	.271
	Worker/student	2.07 (0.949)		
Total		2.17 (0.950)		

*Note*. Bold indicates statistically significant difference after applying the Bonferroni correction. BMI = body mass index.

There was no statistically significant correlation between the level of knowledge and the level of attitudes (*r* = –.064, *p* > .05).

Results from the generalised linear model indicated that the scientific area of study, sex of students and the attitudes towards self-medication had a statistically significant effect in the measure of self-medication practices. Thus, attending engineering sciences (*β* = .718, 95% CI: 1.373–3.069, *p* < .001), being female (*β* = .866, 95% CI: 1.700–3.327, *p* < .001) and having negative attitudes towards self-medication (*β* = .367, 95% CI: 1.227–1.698, *p* < .001) predict the adoption of those practices (Table 5).

**Table 5.** Generalised linear model predicting self-medication.

	β	*SE*	χ^2^ Wald	*df*	*p*	Exp (β)	95% CI	
Intercept	–.848	.2720	9.721	1	.002	0.428	0.251	0.730
**Scientific area**								
Engineering sciences	.718	.2048	12.298	1	**.000**	2.051	1.373	3.064
Exact and natural sciences	.401	.2284	3.088	1	.079	1.494	0.955	2.337
Judicial and economic sciences	.346	.2433	2.019	1	.155	1.413	0.877	2.276
Social and human sciences	0	–	–	–	–	1	–	–
**Sex**								
Male	0					1		
Female	.866	.1713	25.593	1	**.000**	2.379	1.700	3.327
Knowledge about self-medication	.158	.0835	3.600	1	.058	1.172	0.995	1.380
Attitudes towards self-medication	.367	.0830	19.533	1	.000	1.443	1.227	1.698

*Notes*. 95% CI = 95% confidence intervals; AIC = 904.432; χ^2^ (6) = 54.359, *p* < .001. Bold indicates statistically significant difference from reference group after applying the Bonferroni correction.

## Discussion

In our study, more than half of college students reported self-medication practices in the last 12 months. This corroborates the findings of a study conducted in 2014 in Portugal, where it was found that 58.7% of incoming students self-medicated (Mendes & Lopes, 2014).

The prevalence of consumption of medicines without prescription in higher education varies by geographical area, so in previous studies in the Middle East region it was shown that university students engaged more frequently in self-medicating behaviour than the subjects analysed in our study (Palestine – 98%, Sawalha, 2008; Kuwait – 70.4%, Mitra et al., 2019; New Delhi – 85.4%, Adhikary et al., 2014; Saudi Arabia – 73%, Alshawi et al., 2018 and 65.58%, Mustafa & Rohra, 2017; India – 84.0%, Kumar et al., 2013; Nepal – 83.3%, Karmacharya et al., 2018 and 81.9%, Gyawali, 2015). In this region studies were conducted in some countries which also identified a similar prevalence of this behaviour when compared to Portuguese university students (Egypt – 55%, El-Ezz & Ez-Elarab, 2011; Bangladesh – 54.5%, Idris et al., 2016; Kingdom of Bahrain – 44.8%, James et al., 2006). African university students were those with the lowest self-medication rate, according to the study by Abay and Amelo (2010) in Ethiopia, compared to students from Middle Eastern and European countries (Italy –69.2%, Garofalo et al., 2015; Greece – 44.6%, Skliros et al., 2010; Serbia – 79.9%, Lukovic et al., 2014). These statistical data show that the economic situation of a country may influence self-medicating practices among university students, according, for example, to the research conducted by Xu and colleagues (2019). In this sense, according to Bennadi (2014), correct practices of self-medication may constitute an important replacement for formal health systems, providing an opportunity for access to immediate healthcare.

Analgesics have been reported as the group of medicines most commonly used by college students, in a similar fashion to other studies (Bennadi, 2014; Hughes et al., 2001; James et al., 2006; Karmacharya et al., 2018; López-Cabra et al., 2016; Lukovic et al., 2014; Mehta & Sharma, 2015), followed by vitamins (González-Castillo et al., 2019; Lukovic et al., 2014; Mir, 2015), while tranquilisers were rarely used (Mir, 2015).

The high prevalence of self-medication among university students may be related to: the perception that they possess enough and adequate knowledge; the wrongful perception that an illness with non-severe symptoms does not produce serious consequences in case of self-medication (Mehta & Sharma, 2015); the belief that self-medicating provides quick relief from pain and disease (Tuyishimire et al., 2019).

Sex and age of respondents are among the variables that are often associated with self-medication (Carrasco-Garrido et al., 2014). By analysing our results, we conclude that self-medication was significantly higher among females when compared to males, which is consistent with what was reported in the literature (Garofalo et al., 2015; Helal & Abou-Elwafa, 2017; James et al., 2006). Nevertheless, contrary to what was identified in several studies, we found that increased university performance seems to have a significant positive impact on self-medicating behaviours (Alshawi et al., 2018; Garofalo et al., 2015; Helal & Abou-Elwafa, 2017; James et al., 2006; Kasulkar & Gupta, 2015; Klemenc-Ketis et al., 2010), and we did not find any significant differences in prevalence of self-medicating behaviour among incoming and finalist students. These results were similar to those obtained in the study conducted by Shankar and colleagues (2016).

Regarding knowledge about self-medication, there was a low level of knowledge, corroborating the findings of other international studies with university students (Gyawali, 2015; James et al., 2006; Mitra et al., 2019), in particular medical courses (Shankar et al., 2016). However, after reviewing the existing literature on this subject, other studies have been identified in which university students displayed a good level of knowledge about self-medication (Karmacharya et al., 2018; Mehta & Sharma, 2015). As expected (Adhikary et al., 2014; Corrêa da Silva et al., 2012; Galato et al., 2012; Mustafa & Rohra, 2017), the level of knowledge about self-medicating is directly connected with practices of self-medication, or, in other words, the students who reported having engaged in self-medicating practices in the last 12 months were the ones who presented a higher level of knowledge on the topic. This means that the more students know about self-medication, including its potential consequences, the more likely they are to engage it in, perhaps due to a wrongful perception that there are more benefits than hazards directly related to this practice. However, this association was nullified in our study after we applied the generalised linear model, which results in the conclusion that level of knowledge about self-medication is not necessarily a predictive factor of a greater likelihood of engaging in self-medicating practices.

Girls had a higher knowledge score than boys and students in the first year had more knowledge than students in the third year. In the study by Mitra and collaborators (2019), females were also shown to possess a higher knowledge score, that dictates to some degree of perfection the knowledge level, than males. However, students belonging to higher academic year groups scored higher knowledge levels than those of lower academic year groups. Other studies do indeed find a correlation between the year of study and the level of knowledge about self-medication, which we interpret as indicating that knowledge increases with the level of education (Gyawali, 2015; Shankar et al., 2016). This emphasises the importance of improving the undergraduate curriculum by providing adequate access to content that deals specifically, in depth and scientifically with health knowledge to college students, making it available to them in a generalised manner, as accessible as possible, because self-medication was adequate in only 14.2% of cases (James et al., 2006).

In accordance with other studies (James et al., 2006; Mehta & Sharma, 2015), the attitude towards self-medication was positive, meaning students thought that it was not good to self-medicate. Many students have correctly understood that it is inadvisable to use non-prescription medicines for a short time and use previously prescribed medications to treat the same symptoms. According to the generalised linear model, attitudes regarding the use of medication without prescription are one the factors that contribute to the process of self-medicating. Therefore, students who favoured self-medication saying or implying that it was acceptable show a higher prevalence of the practice of self-medication in the previous year. As we previously pointed out, corroborating scientific literature, being female increases the likelihood of self-medicating by 2.37 times when compared to men. This finding may be justified, in the present study, by the fact that women possess a higher level of knowledge about self-medication than men, but it can also be, at least in part, attributed to social and cultural factors, such as the cautious and vigilant nature of women (Mitra et al., 2019), or biological ones, for example, in the case of menstrual pain (Donkor et al., 2012; Mogil, 2012).

We are unaware of any scientific study that covers the differences between self-medication and the scientific area of study, with the exception of students in the health area vs. those in other areas. Nonetheless, our study has shown that being a student in the area of engineering sciences increases the likelihood of engaging in self-medicating practices, when compared to students belonging to social and human sciences. This predictive factor for self-medication may be related to the fact that university courses in the area of engineering sciences are quite demanding in academic terms, which would explain why future engineers were those that consumed more vitamins/food supplements in the last year.

This study, and any usage of it, must take into account the limitations which can affect the interpretation of findings and, as a consequence, having an impact on its final conclusions. Restricting the study to a single university limits generalisability to the total population of university students in Portugal. In addition, recall bias may be a major disadvantage as students were asked to provide information on self-medication during the previous year, and so, the results subject to analysis were found to be highly dependent on the students’ recollection ability and their honesty and truthfulness, which means that the results may not reflect in a perfect manner how students behave. The present study has not taken into consideration the specific periods of time in which students self-medicated (for example, when studying and preparing for exams). In this sense, future studies should consider the importance of deepening the understanding of the reasons behind self-medication behaviours and practices in the academic context. Another further limitation that must be considered is that the study has not made any distinction between the use of OTC medication in self-medicating practices and previously prescribed medicine, and we suspect that this might have been confusing for the questioned students. One final limitation should be considered. The absence of information from courses belonging to the area of health sciences, although a methodological choice, does not mean we have at all dismissed the importance of analysing the knowledge, attitudes and prevalence of self-medication practices in this group in future research, since the meta-analysis study carried out by Xu and colleagues (2019) has shown a significantly higher prevalence of self-medication with antibiotics among medical students when compared to non-medical students.

## Conclusions

This cross-sectional study shows that self-medication practices were very common among university students in Portugal. Knowledge about self-medication has been shown to be poor and the level of attitude towards self-medication found to be positive. Self-medication in the research sample is connected with female students, those who belong in the scientific area of engineering sciences and those with a highly negative level of attitudes towards self-medication.

Given these results, there is a great probability of occurrence of irresponsible and inadequate use of self-medication among university students due to the low knowledge they revealed. Therefore, the recommendation to increase knowledge about the adverse effects of OTC medications and to increase awareness about the importance of educational programmes in this field becomes an obvious one, but it cannot be the only way. Multiple actions of intervention need to be adopted in order to solve that problem, including not only changes in knowledge levels and personal attitudes or behaviours, but also changes made to the level and type of support given to national policies and laws.
